# Impact of visceral adiposity index on cognitive impairment and cognitive trajectories in Chinese middle-aged and older adults

**DOI:** 10.3389/fpubh.2025.1612801

**Published:** 2025-08-01

**Authors:** Siran Chen, Mengqi Zhou, Lin Han, Rui Ma, Xiaoyue Jiang, Ziyi Yang, Yuling Du, Yanfang Yang

**Affiliations:** ^1^Department of Epidemiology and Health Statistics, West China School of Public Health and West China Fourth Hospital, Sichuan University, Chengdu, Sichuan, China; ^2^Department of Neurology, West China Fourth Hospital, Sichuan University, Chengdu, Sichuan, China

**Keywords:** visceral adiposity index, visceral obesity, cognitive function, cognitive trajectory, CHARLS

## Abstract

**Introduction:**

The association between the visceral adiposity index (VAI) and cognitive function in middle-aged and older Chinese adults is not well understood. The “obesity paradox”—where obesity appears to be linked with better health outcomes—has also been observed. This study aims to clarify the association by using data from a nationally representative longitudinal survey.

**Methods:**

This study analyzed five waves of data from the China Health and Retirement Longitudinal Study (CHARLS) from 2011 to 2020. The Cox proportional hazards regression model was used to assess the effect of VAI on the occurrence of cognitive impairment. Additionally, cognitive trajectories over the study period were identified using group-based trajectory modeling (GBTM), and the association between VAI and cognitive trajectories was further analyzed through multinomial logistic regression.

**Results:**

A total of 5,637 participants aged ≥45 years were included, of whom 46.6% were women. The risk of cognitive impairment was lower in participants with higher VAI scores (Q3: HR = 0.79, 95% CI: 0.67–0.94; Q4: HR = 0.83, 95% CI: 0.70–0.98). Cognitive trajectories over the 9-year period were categorized into four groups based on cognitive Z-scores: “high and stable” (*n* = 621, 12.6%), “middle and stable” (*n* = 2,157, 36.7%), “low and stable” (*n* = 1,856, 32.8%), and “low and decline” (*n* = 1,003, 17.9%). After adjusting for demographic and health-related variables, participants in the highest VAI quartile (Q4) had a significantly lower likelihood of experiencing cognitive decline (adjusted OR = 0.67, 95% CI: 0.48–0.93).

**Conclusion:**

Greater visceral adiposity was associated with a lower risk of developing cognitive impairment and a more favorable cognitive trajectory over time.

## 1 Introduction

Dementia is currently the seventh leading cause of death and one of the primary sources of disability and dependency among older people globally (1). With the aging of the global population, the number and proportion of people over 60 years old is increasing (2). China has the largest older population in the world. According to data from the seventh national census, there are currently 260 million individuals aged 60 and older in the country, making up 18.7% of the total population (3). As the older population grows, so too does the number of individuals affected by dementia. In 2020, more than 55 million people worldwide were living with dementia, and this number is expected to nearly triple by 2050, with China accounting for approximately 25% of the global total (4, 5). Given the lack of effective treatment for dementia, prevention is a crucial focus (6). Cognitive decline is a distinctive feature of dementia, so identifying risk factors for cognitive decline plays an important role in delaying and preventing dementia (7, 8). Numerous risk factors for cognitive decline have been identified, including age, education level, smoking, physical activity, obesity, and the presence of conditions like hypertension, diabetes, and hyperlipidemia (9). Among these, the association between obesity and cognitive function remains particularly contentious.

With rapid economic development and changes in lifestyles, obesity has become increasingly prevalent and now poses a significant public health threat. A 2020 report from the National Health Commission of the People’s Republic of China revealed that over half of Chinese adults are overweight or obese (10). Obesity has long been linked to a higher risk of various chronic conditions, such as hypertension, diabetes, and cardiovascular disease (11). Similarly, obesity is recognized as a risk factor for cognitive decline and dementia (12–14). Several biological mechanisms may explain the link between obesity and cognitive decline, such as obesity triggering neuroinflammation, insulin resistance, and brain mitochondrial dysfunction, which can impair hippocampal neuroplasticity (15). However, some studies suggest that obesity might be associated with better cognitive function, a phenomenon known as the “obesity paradox” (16–19). One proposed mechanism is that leptin, secreted by adipose tissue, enhances synaptic plasticity and modulates hippocampal function-processes critical for maintaining cognitive health. Additional mechanisms may include increased secretion of irisin and estrogen, improved nutritional status and energy reserves, as well as increased brain volume and enhanced cognitive reserve (20). These studies of the “obesity paradox” often rely on body mass index (BMI) or waist circumference to measure obesity, but these indicators fail to account for fat distribution, making them less reliable (21, 22). Individuals with the same BMI can have markedly different body fat compositions. Where fat is stored in the body can influence health outcomes in distinct ways. In middle-aged and older adults, obesity tends to concentrate around the abdomen (23). However, waist circumference—a common measure of abdominal fa—cannot differentiate between subcutaneous fat and visceral fat (24). The visceral adiposity index (VAI), which combines BMI, waist circumference, triglycerides, and high-density lipoprotein cholesterol, offers a more accurate measure of fat distribution and function (25). Currently, there is limited research exploring VAI and cognitive function in Chinese middle-aged and older populations by prospective design. Zeng et al. reported that higher levels of visceral fat were linked to a slower rate of cognitive decline (26). However, their study was limited by a short follow-up period and a small sample size. Most existing research on the relationship between visceral adiposity and cognitive function relies on MRI or CT scans to assess fat distribution. While these imaging techniques provide precise measurements, they are rarely used in large, long-term studies due to their high cost, time demands, and potential radiation exposure. As a result, many studies in this field are cross-sectional with limited sample sizes, and their findings have been inconsistent (27–29).

Therefore, this study examined the effects of visceral adiposity on cognitive function using data from the China Health and Retirement Longitudinal Study (CHARLS). In addition, we identified cognitive function trajectories and explored the association between visceral adiposity and these trajectories. We hypothesize that higher levels of visceral fat are linked to a greater risk of cognitive decline compared to lower levels and our longitudinal analysis will reveal distinct trajectories of cognitive function within the study population.

## 2 Methods

### 2.1 Study design and participants

CHARLS project is designed to gather high-quality data on households and individuals aged 45 and older across China. This dataset provides valuable insights into population aging and supports interdisciplinary research on aging-related issues. CHARLS national baseline survey was conducted in 2011 using the multi-stage probability to proportional to size (PPS) sampling method, covering 150 county-level units and 450 village-level units, with a sample size of approximately 17,000 individuals from 10,000 households (30). To date, CHARLS has released data from five national surveys, including the initial baseline survey (2011), the first (2013), the second (2015), the third (2018), and the fourth (2020) follow-up surveys. These datasets are available for download on the CHARLS website at https://charls.pku.edu.cn/. The CHARLS project was approved by the Biomedical Ethics Committee of Peking University, with all participants providing informed consent (approval number: IRB00001052-11015).

**Figure 1:**
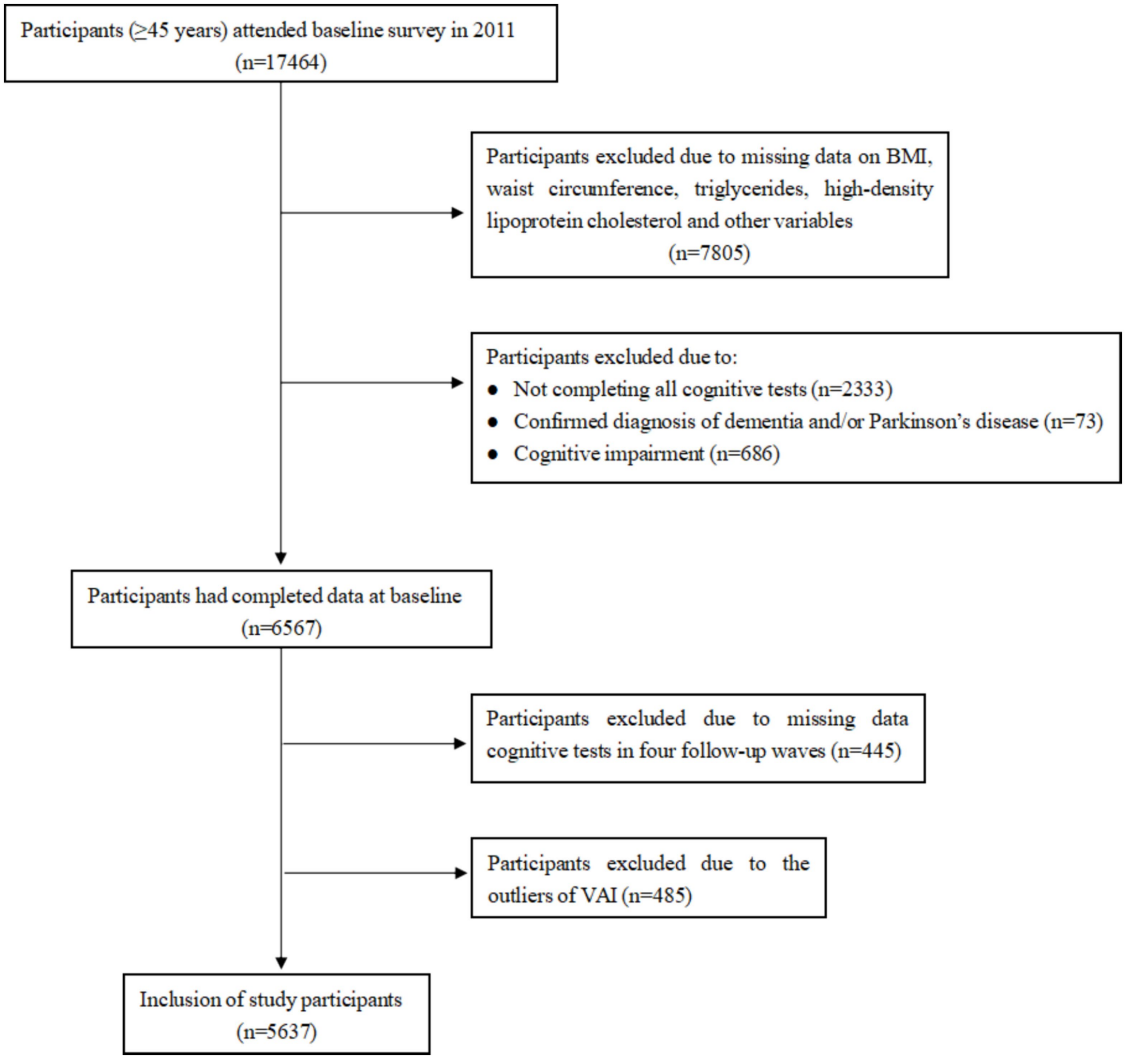
Flowchart of participant selection for the study.

We utilized data from the baseline and follow-up surveys of CHARLS, conducted in 2011, 2013, 2015, 2018, and 2020. A total of 17,708 individuals from 10,257 households were successfully interviewed for the 2011 baseline CHARLS survey, of which 17,464 were within the representative sample age of 45 years or older. We excluded 10,897 individuals due to (1) no information on BMI, waist circumference, triglycerides, high-density lipoprotein cholesterol, and other variables (*n* = 7,805), (2) not completing all cognitive tests (*n* = 2,333), confirmed diagnosis of dementia and/or Parkinson’s disease (*n* = 73) and cognitive impairment (31) (defined as a cognitive score < 9 [1.5 times the standard deviation below its mean], *n* = 686). The baseline survey included 6,567 participants. In the follow-up surveys, we excluded the participants due to missing data on cognitive assessment (*n* = 445). To ensure data quality, any participants with abnormal values for the VAI, based on Tukey method (above or below 1.5 times the inter-quartile range), were also excluded (*n* = 485), which resulted in 5,637 eligible individuals. The detailed flow chart of participant selection is shown in Figure 1.

### 2.2 Calculation of VAI

The VAI, a measure of visceral adiposity deriving from anthropometric and metabolic parameters, could completely reflect visceral fat dysfunction (32). The VAI was calculated using distinct formulas for males and females. For men, the formula is: VAI = [WC (cm)/(39.68 + 1.88 × BMI (kg/m^2^))] × (TG (mmol/L)/1.03) × (1.31/HDL (mmol/L)), For women, the formula is: [WC (cm)/(36.58 + 1.89 × BMI (kg/m^2^))] × (TG (mmol/L)/0.81) × (1.52/HDL (mmol/L)). In these formulas, WC represents waist circumference, BMI is body mass index, TG stands for triglycerides, and HDL indicates high-density lipoprotein cholesterol. After calculating the VAI values, they were categorized into quartiles for analysis. These quartiles were defined as follows: Quartile 1 (Q1, <0.84), Quartile 2 (Q2, 0.84–1.37), Quartile 3 (Q3, 1.37–2.27) and Quartile 4 (Q4, >2.27).

### 2.3 Assessment of cognitive score

Participants received face-to-face assessments of four cognitive domains: orientation, calculation, memory, and drawing. Orientation and calculation were assessed using the Telephone Interview for Cognitive Status (TICS). The orientation task required participants to identify the current year, month, day, week, and season, while the calculation task involved subtracting 7 from 100 consecutively for five times. Each correct response earned one point, with a total possible score ranging from 0 to 10. Memory was evaluated through immediate and delayed recall. Participants were read a list of 10 words, and their recall was tested immediately and after a short delay. The total memory score was 20 points, with one point for each word. In addition, participants were asked to accurately redraw a figure presented by the interviewer, with one point awarded for a correct reproduction. The total score of cognitive was defined as the sum scores of orientation (5 points), computation (5 points), memory (20 points), and drawing (1 point), resulting in 31 points (33). A lower score indicated worse cognitive performance.

### 2.4 Covariates

Based on previous studies, we controlled potential confounding covariates in the analysis. Demographic characteristics included age (45–59, or ≥60 years), sex (male or female), education (elementary school or below, secondary school, or college and above), residence (rural or city), and marital status (married, or unmarried). Health-related factors included ever smoking (yes or no) and ever drinking (yes or no).

### 2.5 Statistical analysis

In the present study, continuous variables were presented as means ± standard deviations (SDs), and categorical variables were presented as numbers (percentages). Differences between continuous variables were assessed using analysis of variance (ANOVA), while categorical variables were compared using Chi-square tests.

We use the Cox proportional hazards regression models to examine the association between VAI and cognitive impairment. Results were presented as the hazard ratios (HR) with 95% CIs. The examination date of the baseline examination (2011) was considered as time zero for the survival model and the endpoint was defined as the occurrence of cognitive impairment (defined as 1.5 times standard deviation below the mean value). Moreover, the proportional hazards (PH) assumption was satisfied based on Schoenfeld residual testing.

Trajectories of cognitive scores were identified by group-based trajectory modeling (GBTM) to describe the developmental course of cognitive scores over time (34). We first used a multiple regression equation adjusting for age, sex, and education to obtain the predicted cognitive scores, and then, we used the following equation to calculate the adjusted Z scores: $Z=\frac{Y-\overset{¯\prime }{Y}}{\text{RMSE}}$ , where Y is the raw cognitive score, $\overset{¯\prime }{Y}$ is the predicted population mean score, and RMSE is the root mean square error of the regression equation (35). The transformed cognitive Z scores were used in analyses. The optimal number of trajectory subgroups was selected by Bayesian information criteria (BIC), Akaike’s information criterion (AIC), and the average posterior probability. Furthermore, the average posterior probability of trajectory was greater than or equal to 0.7, and the membership in each trajectory was at least 5% (34). Subsequently, the multinomial logistic regression model was used to estimate the effects of the VAI on the different cognitive trajectories.

The following variables were adjusted in all models to control for potential confounding factors: demographic variables (age, sex, education level, residence, and marital status) and health-related factors (smoking status and drinking status). The GBTM analysis was conducted using the Traj plugin in Stata 17 software. Other statistical analyses were performed using R version 4.4.1, with a two-tailed *p*-value of less than 0.05 indicating statistical significance.

## 3 Results

### 3.1 Baseline characteristics

A total of 5,637 participants were included in this study, of which 47.4% were aged 60 years or above and 46.6% were female. Participants were categorized into four groups based on their visceral adiposity index quartiles: Q1 (<0.84), Q2 (0.84–1.36), Q3 (1.36–2.27), and Q4 (>2.27). As shown in Table 1, the corresponding baseline cognitive scores were, respectively, 16.71 ± 3.83, 16.85 ± 3.89, 16.87 ± 3.82, and 17.19 ± 3.91, with statistically significant differences among the four groups (*p* = 0.009). In addition, significant differences in age, gender and residence distribution between the groups could be observed (*p* = 0.005 for age, *p* < 0.001 for gender, *p* < 0.001 for residence). Regarding the variables of educational level and marital status, there were no significant differences between the VAI groups. Finally, health-related factors, including smoking and drinking, showed significant differences between the groups (*p* < 0.001 for both), and participants in the high VAI group were less likely to smoke or consume alcohol.

**Table 1:** Characteristics of participants by VAI quartile.

Characteristic	Overall (*n* = 5,637)	Q1 (*n* = 1,410)	Q2 (*n* = 1,409)	Q3 (*n* = 1,409)	Q4 (*n* = 1,409)	*p* value
Age, *n* (%)						0.005
45–59	2,966 (52.6)	684 (48.5)	753 (53.4)	762 (54.1)	767 (54.4)	
≥60	2,671 (47.4)	726 (51.5)	656 (46.6)	647 (45.9)	642 (45.6)	
Sex, *n* (%)						<0.001
Male	3,008 (53.4)	1,035 (73.4)	800 (56.8)	643 (45.6)	530 (37.6)	
Female	2,629 (46.6)	375 (26.6)	609 (43.2)	766 (54.4)	879 (62.4)	
Education, *n* (%)						0.434
Elementary school or below	3,411 (60.5)	852 (60.4)	843 (59.8)	851 (60.4)	865 (61.4)	
Secondary school	2,122 (37.6)	534 (37.9)	531 (37.7)	539 (38.3)	518 (36.8)	
College and above	104 (1.8)	24 (1.7)	35 (2.5)	19 (1.3)	26 (1.8)	
Residence, *n* (%)						<0.001
Rural	4,437 (78.7)	1,157 (82.1)	1,128 (80.1)	1,108 (78.6)	1,044 (74.1)	
City	1,200 (21.3)	253 (17.9)	281 (19.9)	301 (21.4)	365 (25.9)	
Marital status, *n* (%)						0.536
No	523 (9.3)	131 (9.3)	118 (8.4)	140 (9.9)	134 (9.5)	
Yes	5,114 (90.7)	1,279 (90.7)	1,291 (91.6)	1,269 (90.1)	1,275 (90.5)	
Smoking, *n* (%)						<0.001
No	3,216 (57.1)	610 (43.3)	769 (54.6)	897 (63.7)	940 (66.7)	
Yes	2,421 (42.9)	800 (56.7)	640 (45.4)	512 (36.3)	469 (33.3)	
Drinking, *n* (%)						<0.001
No	3,550 (63.0)	686 (48.7)	872 (61.9)	974 (69.1)	1,018 (72.2)	
Yes	2,087 (37.0)	724 (51.3)	537 (38.1)	435 (30.9)	391 (27.8)	
Cognitive score, mean (SD)	16.90 (3.86)	16.71 (3.83)	16.85 (3.89)	16.87 (3.82)	17.19 (3.91)	0.009

SD, standard deviation.

### 3.2 Association between VAI and cognitive impairment

A total of 1,068 (18.95%) participants developed cognitive impairment during the 9-year follow-up. The risk of cognitive impairment was significantly lower in the higher VAI groups compared to the Q1 group (Q3: HR = 0.79, 95% CI: 0.67–0.94; Q4: HR = 0.83, 95% CI: 0.70–0.98; Table 2). This association persisted after adjusting for demographic and health-related variables (Q3: HR = 0.78, 95% CI: 0.66–0.94; Q4: HR = 0.80, 95% CI: 0.67–0.96; Table 2). The Kaplan–Meier curves showed a lower incidence of cognitive impairment in the group with a higher VAI (Supplementary Figure S1). In addition, those with cognitive impairment were more likely to be older, female, less educated, live in rural areas, and unmarried (Supplementary Figure S2).

**Table 2:** Hazard ratios with cognitive impairment by VAI quartile.

VAI	Event (%)	Model 1		Model2	
		HR (95%CI)	*p*	HR (95%CI)	*p*
Q1	290 (20.57)	Ref		Ref	
Q2	290 (20.58)	0.97 (0.82, 1.14)	0.690	0.97 (0.83, 1.15)	0.763
Q3	239 (16.96)	0.79 (0.67, 0.94)	0.008	0.78 (0.66, 0.94)	0.007
Q4	249 (17.67)	0.83 (0.70, 0.98)	0.027	0.80 (0.67, 0.96)	0.014

Model 1 was conducted without any adjustment; Model 2 was adjusted for age, sex, education, residence, marital status, smoking status, and drinking status.

HR, hazard ratio; CI, confidence interval; Ref, reference; VAI, visceral adiposity index.

### 3.3 Association between VAI and cognitive trajectories

**Figure 2:**
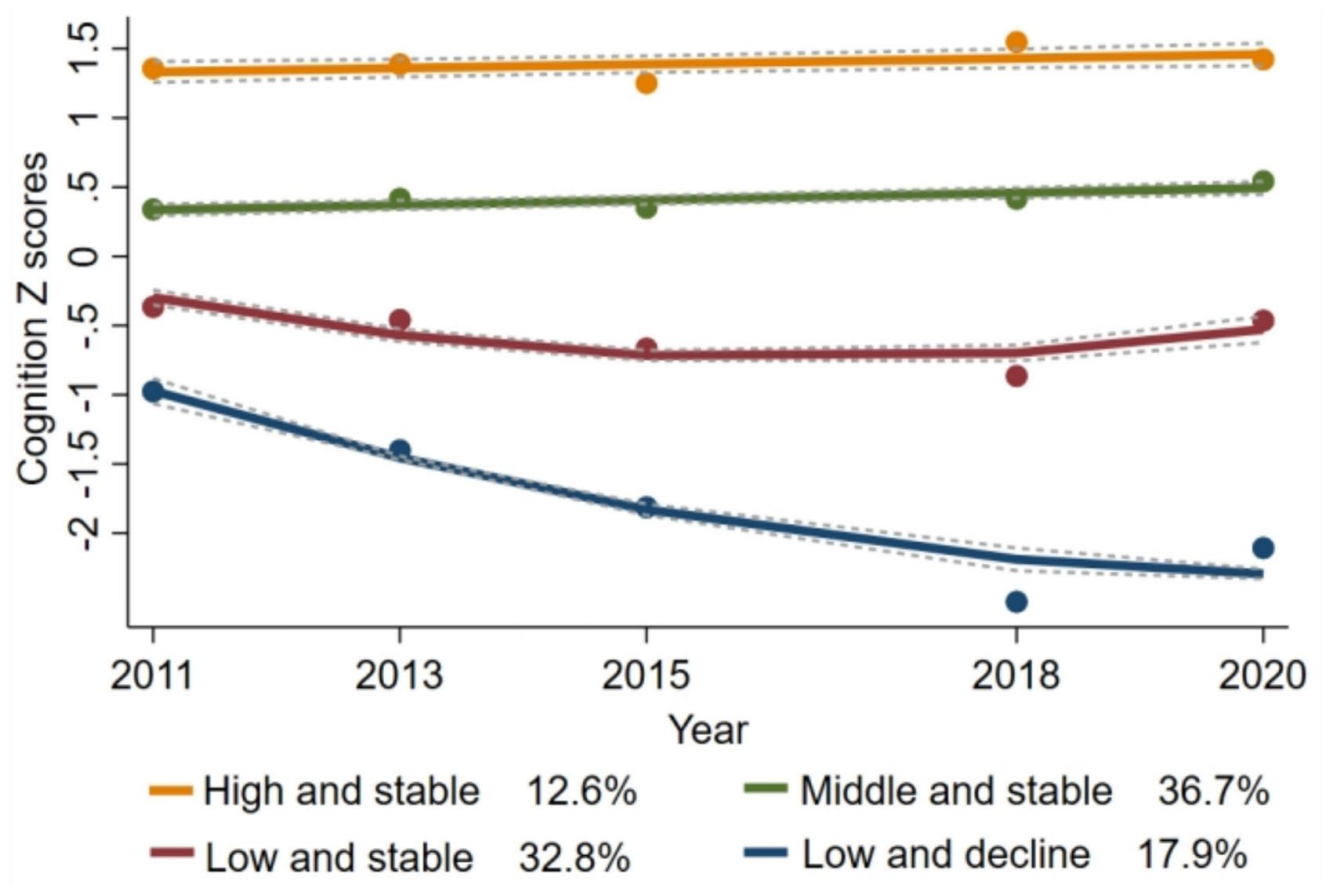
Trajectories of cognitive scores from 2011 to 2020.

Various models were tested, and their parameters are detailed in Supplementary Table S1. Ultimately, a four-group trajectory model was identified as the best fit, based on the lowest Bayesian information criterion (BIC = −34,058.01), the average posterior probabilities exceeding 0.7, and subgroup proportions greater than 5%. Supplementary Table S2 summarizes the maximum likelihood estimates for the final four groups of trajectories. The four group trajectories for cognitive scores are shown in Figure 2: (1) “high and stable” (*n* = 621, 12.6%); (2) “middle and stable” (*n* = 2,157, 36.7%); (3) “low and stable” (*n* = 1,856, 32.8%); and (4) “low and decline” (*n* = 1,003, 17.9%). Supplementary Table S3 shows the baseline characteristics of participants with different cognitive trajectories. Participants in the “low and decline” trajectory group were more likely to be older, female, have lower education attainment, reside in rural areas, and be unmarried than participants in the “high and stable” group.

Using the “high and stable” trajectory as a reference, multinomial logistic regression analyses showed that the Q4 group was associated with a decreased likelihood of having a cognitive decline trajectory compared with the Q1 group (adjusted OR = 0.67, 95% CI: 0.48–0.93; Table 3). Further analysis stratified by age and sex revealed similar results (both *p* > 0.05 for interaction, Supplementary Figure S3).

**Table 3:** Multinomial logistic regression analysis of the relationship between VAI quartile and cognitive trajectories.

VAI	Middle and stable^a^		Low and stable^a^		Low and decline^a^	
	OR (95% CI)^b^	*p*	OR (95% CI)^b^	*p*	OR (95% CI)^b^	*p*
Q1	Ref		Ref		Ref	
Q2	1.12 (0.85, 1.47)	0.425	1.08 (0.81, 1.15)	0.608	1.00 (0.72, 1.39)	0.988
Q3	0.98 (0.75, 1.28)	0.867	0.91 (0.68, 1.21)	0.500	0.78 (0.56, 1.09)	0.063
Q4	0.95 (0.73, 1.25)	0.727	0.76 (0.57, 1.02)	0.145	0.67 (0.48, 0.93)	0.016

Ref, reference; OR, odds ratio; CI, confidence interval; VAI, visceral adiposity index.

a Reference group was the high and stable group.

b Adjusted for age, sex, education, residence, marital status, smoking status, and drinking status.

### 3.4 Sensitivity analyses

To ensure greater stability in trajectory analysis, we conducted a sensitivity analysis focusing on participants who had completed four or more cognitive assessments. Among these participants (*n* = 3,966), the identified cognitive trajectories were consistent with those observed in the main analyses: (1) “high and stable” (*n* = 476, 13.1%); (2) “middle and stable” (*n* = 1,643, 39.9%); (3) “low and stable” (*n* = 469, 12.3%); and (4) “low and decline” (*n* = 1,378, 34.6%) (Supplementary Figure S4). Moreover, the associations between VAI and these cognitive trajectories were similar to the primary findings (Supplementary Table S4).

## 4 Discussion

We used a nationally representative sample of middle-aged and older Chinese adults to examine the impact of visceral adiposity on cognitive function. The results indicate that higher VAI scores have a lower risk of cognitive impairment. Additionally, we explored different trajectories of cognitive function over time, identifying four distinct patterns: high and stable, middle and stable, low and stable, and low and decline. Our findings show that high VAI was associated with a better trajectory of cognitive performance.

Obesity is commonly caused by the excessive accumulation of body fat, and its potential link to cognitive function has long been a subject of research. There is evidence that obesity is associated with cognitive decline and an increased risk of dementia (12–14). One proposed mechanism is that obesity, as a known vascular risk factor, may lead to blood–brain barrier dysfunction and reduced cerebral blood flow, further damaging brain tissue and impairing cognitive functions (36). However, the association between obesity and cognitive function remains controversial. Some studies report that obesity is associated with better cognitive performance (16–19). This inconsistency may be due to variations in study samples, obesity assessment methods, cognitive function measures, follow-up duration, and adjustment for potential confounders. In our study, we focused on the visceral adiposity index (VAI), a measure of visceral fat, to examine its association with cognitive function. While visceral adiposity has been linked to an increased risk of insulin resistance, metabolic syndrome, and cardiometabolic diseases (37), our findings suggest that higher VAI scores are unexpectedly associated with a lower risk of cognitive impairment. Additionally, we found that older age, female gender, lower education levels, unmarried status, and rural living were factors that increased the risk of cognitive impairment, consistent with previous studies (38, 39).

This study also explored cognitive function trajectories over time in a middle-aged and older population using group-based trajectory modeling (GBTM), emerging four main patterns, including high and stable, middle and stable, low and stable, and low and decline. Previous research has identified varying numbers of cognitive trajectory categories, such as two (40, 41), three (42, 43), four (44, 45), or five (46, 47), which can be attributed to differences in study populations, duration of follow-up, cognitive measurement methods and statistical models employed. Our analysis revealed significant differences in baseline cognitive function across the four trajectory groups. Most individuals maintained relatively stable cognitive scores as they aged, about 17.9% showed signs of declining cognitive function, aligning with findings from prior research (48–50). Furthermore, the trajectory analysis yielded that higher VAI scores were less likely to follow a cognitive decline trajectory, supporting similar conclusions in those described above.

The results from the Cox proportional hazards regression model and analysis of cognitive trajectories suggest that visceral adiposity is associated with better cognitive function. Several mechanisms may underlie this association. First, evidence from cellular, animal, and human studies has demonstrated that leptin, a hormone secreted by adipose tissue, has neuroprotective effects and may help slow cognitive decline (51). Another potential mechanism involves irisin, a myokine produced by adipose tissue, muscle, and the hippocampus. Research in both animals and humans indicates that irisin can regulate expression of brain-derived neurotrophic factor (BDNF) and improve neuronal synaptic plasticity, which could positively affect cognitive function (52). Third, visceral fat is positively correlated with estrogen levels, and estrogen plays a key role in supporting cognitive function (53, 54). For example, estrogen interacts with cell membrane receptors to activate protein kinases, which stimulate the production of specific proteins. This process drives structural changes at existing synapses as well as synaptogenesis, ultimately enhancing memory formation, consolidation, and retrieval (55). Furthermore, obesity is often accompanied by comorbidities such as hypertension, diabetes, and dyslipidemia. These conditions are typically managed more aggressively in obese individuals, potentially leading to better overall health and preservation of cognitive function (26). The potential mechanisms outlined above may help explain the protective association between higher VAI and cognitive function. However, due to the ongoing debate surrounding the “obesity paradox” and the fact that these proposed mechanisms have yet to be fully validated, future research with more robust study designs and advanced experimental methods is needed to understand the complex relationship between obesity and cognitive function.

The strengths of this study are that we used a nationally representative 9-year prospective cohort dataset from China and applied a rigorous statistical approach—Group-Based Trajectory Modeling (GBTM)—to fit cognitive trajectories, allowing us to identify different groups of individuals who experience similar patterns of cognitive function over time. This methodological rigor strengthens the reliability and validity of our results. However, there are some limitations. (1) Our sample consists of middle-aged and older adults in China, which may limit the applicability of our findings to other populations. (2) Due to substantial missing data on physical activity, including these variables in the analysis would have significantly reduced the sample size. This could introduce selection bias and compromise the reliability and representativeness of the findings. As a result, physical activity was excluded from the analysis. (3) Our study relied solely on baseline VAI which may prevent us from examining how changes in visceral adiposity over time might relate to cognitive trajectories. (4) Cognitive function was assessed using a single composite score. We did not analyze specific cognitive domains, such as memory or orientation, which may mask associations with VAI. (5) As an observational study, our findings cannot infer causality. Reverse causation is also possible—for example, early cognitive decline could contribute to weight loss and thereby influencing VAI. Future research using designs with stronger causal inference, such as randomized controlled trials or Mendelian randomization, would overcome this limitation.

## 5 Conclusion

Our study found that increased VAI was protective against cognitive impairment in middle-aged and older Chinese adults. Moreover, trajectory analysis also revealed that individuals with higher VAI scores exhibited more favorable cognitive trajectories over time. These findings challenge the traditional view that higher visceral adiposity adversely affects cognitive function. Therefore, they should be interpreted with caution. To confirm these results and explore the underlying mechanisms, future research should include longer-term longitudinal studies and more detailed experimental investigations.

## Data Availability

The datasets presented in this study can be found in online repositories. The names of the repository/repositories and accession number(s) can be found at: http://charls.pku.edu.cn/en.
