# Supplementary material for: Impact of visceral adiposity index on cognitive impairment and cognitive trajectories in Chinese middle-aged and older adults
Source: Front Public Health. 2025 Aug 1;13:1612801. doi: 10.3389/fpubh.2025.1612801 (PMC12354607; doi:10.3389/fpubh.2025.1612801)
Supplement: Supplementary file 1 [file Table_1.DOCX]

**Supplementary Material**

**Contents**

**Ⅰ. Supplementary Figures**

**Figure S1.** Forest plot of multifactorial Cox analysis between VAI quartile and cognitive impairment.

**Figure S2.** Kaplan-Meier plot of the Cox model examining the association between VAI quartile and cognitive impairment.

**Figure S3.** Stratified analysis by age group and sex for the association of associations of VAI quartile with the membership to cognitive trajectory group.

**Figure S4.** Trajectories of cognitive scores from 2011-2020 (N = 3966).

**Ⅱ. Supplementary Tables**

**Table S1.** The main parameters of the model fit in different groups.

**Table S2.** The final four-group trajectory model of cognitive scores from 2011-2020 in CHARLS.

**Table S3.** Characteristics of participants by the cognitive trajectory.

**Table S4.** Multinomial logistic regression analysis of the relationship between VAI quartile and cognitive trajectories (N = 3966)**.**


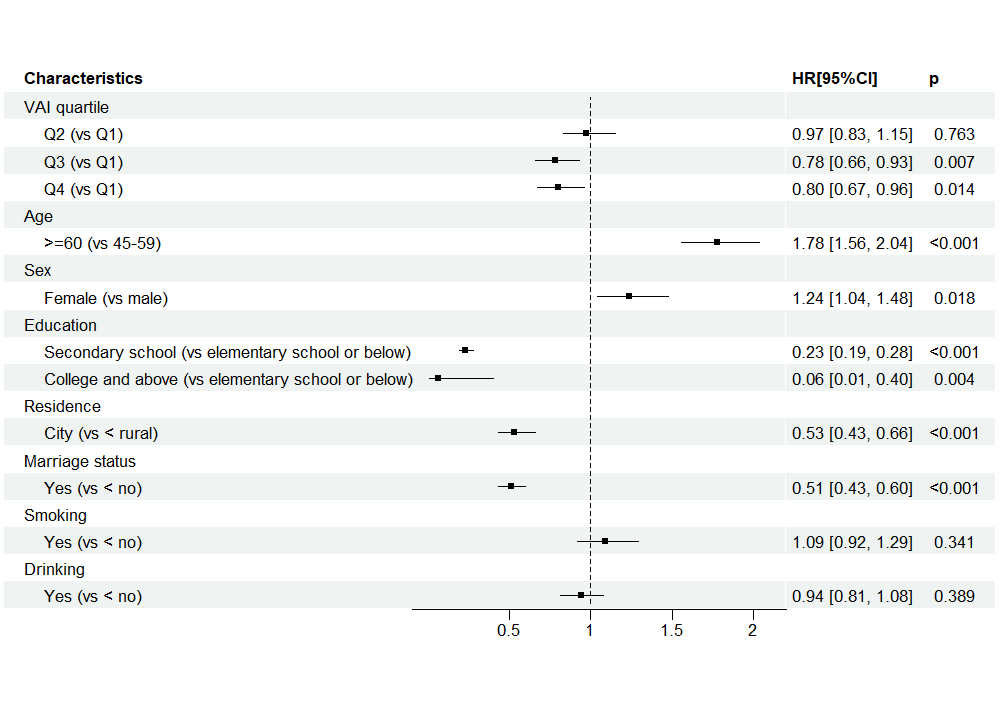


**Figure S1.** Forest plot of multifactorial Cox analysis between VAI quartile and cognitive impairment.

**
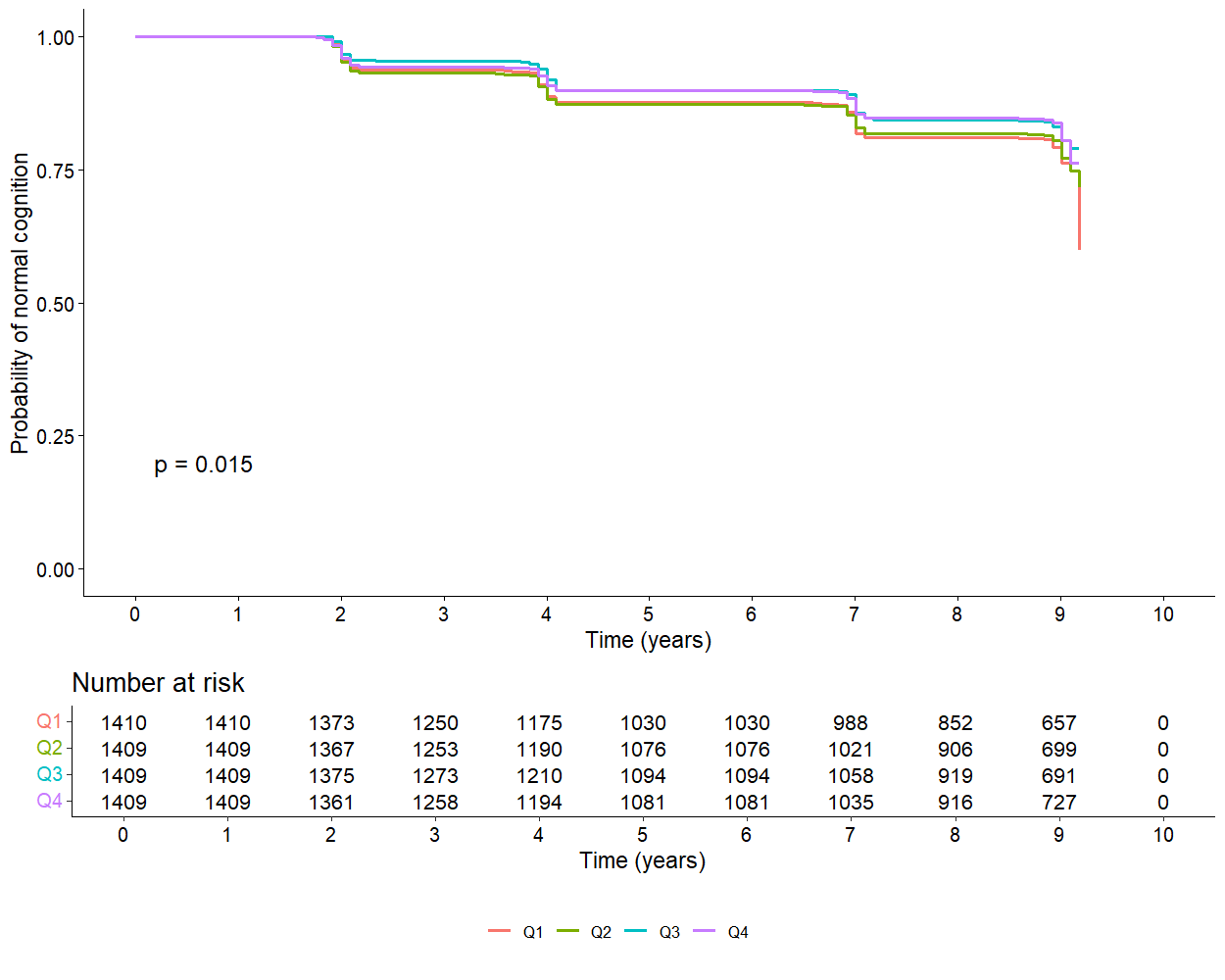
**

**Figure S2.** Kaplan-Meier plot of the Cox model examining the association between VAI quartile and cognitive impairment.

**
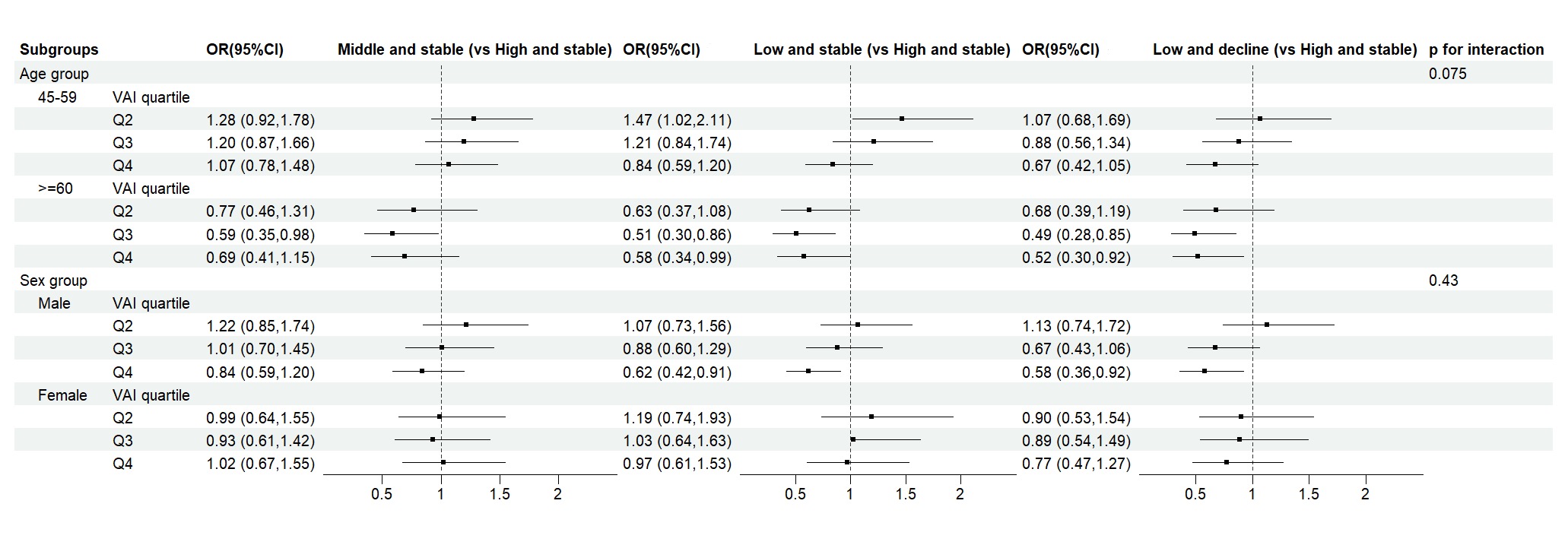
**

**Figure S3.** Stratified analysis by age group and sex for the association of associations of VAI quartile with the membership to cognitive trajectory group.


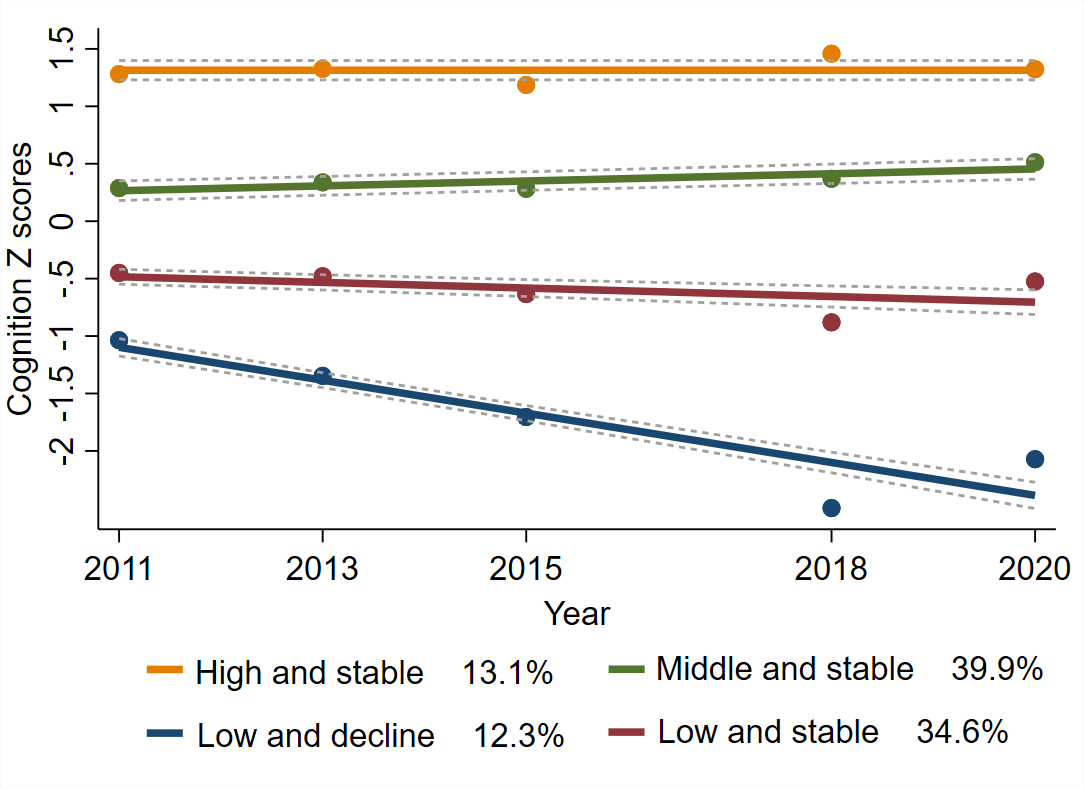


**Figure S4.** Trajectories of cognitive scores from 2011-2020 (N = 3966).Participants were strictly limited to those with cognitive scores available for at least four survey waves.

**Table S1.** The main parameters of the model fit in different groups.

| **Groups** | **Order ^a^** | **AvePP** | | | | **BIC** | **AIC** | **OCC** | | | | **Proportion（%）** | | | |
| --- | --- | --- | --- | --- | --- | --- | --- | --- | --- | --- | --- | --- | --- | --- | --- |
|  |  | **Group 1** | **Group 2** | **Group 3** | **Group 4** |  |  | **Group 1** | **Group 2** | **Group 3** | **Group 4** | **Group 1** | **Group 2** | **Group 3** | **Group 4** |
| **2 groups** | 3 3 | 0.86 | 0.87 |  |  | -36334.46 | -36301.28 | 6.49 | 6.40 |  |  | 49.11 | 50.89 |  |  |
|  | 2 2 | 0.91 | 0.93 |  |  | -35026.74 | -35000.19 | 12.32 | 11.55 |  |  | 45.33 | 54.67 |  |  |
|  | 1 1 | 0.91 | 0.93 |  |  | -35096.68 | -35076.77 | 12.38 | 11.24 |  |  | 45.38 | 54.62 |  |  |
|  | 1 0 | 0.91 | 0.93 |  |  | -35108.70 | -35092.11 | 12.17 | 11.58 |  |  | 45.29 | 54.71 |  |  |
| **3 groups** | 3 3 3 | 0.77 | 0.57 | 0.76 |  | -36280.66 | -36230.88 | 6.47 | 2.44 | 6.68 |  | 32.74 | 34.19 | 33.07 |  |
|  | 2 2 2 | 0.88 | 0.85 | 0.88 |  | -34230.98 | -34191.16 | 24.47 | 5.81 | 19.98 |  | 24.20 | 48.29 | 27.51 |  |
|  | 2 2 1 | 0.88 | 0.85 | 0.88 |  | -34227.51 | -34191.01 | 24.66 | 5.86 | 19.64 |  | 24.26 | 48.37 | 27.37 |  |
|  | 1 0 1 | 0.88 | 0.85 | 0.88 |  | -34296.06 | -34269.51 | 22.83 | 6.14 | 19.10 |  | 24.43 | 48.50 | 27.08 |  |
|  | 0 1 1 | 0.87 | 0.84 | 0.87 |  | -34672.57 | -34646.02 | 21.73 | 5.09 | 19.55 |  | 24.85 | 48.57 | 26.58 |  |
|  | 1 0 0 | 0.88 | 0.86 | 0.87 |  | -34303.92 | -34280.69 | 22.99 | 6.24 | 18.00 |  | 24.52 | 48.73 | 26.74 |  |
| **4 groups** | 3 3 3 3 | 0.41 | 0.68 | 0.70 | 0.42 | -36270.20 | -36203.83 | 2.64 | 5.52 | 5.71 | 2.37 | 25.06 | 24.82 | 25.04 | 25.08 |
|  | 2 2 2 2 | 0.79 | 0.83 | 0.84 | 0.76 | -34067.24 | -34014.15 | 5.90 | 37.20 | 24.50 | 6.59 | 36.93 | 12.76 | 17.70 | 32.61 |
|  | 2 2 2 1 | 0.84 | 0.79 | 0.76 | 0.83 | -34062.95 | -34013.17 | 24.49 | 5.93 | 6.59 | 40.00 | 17.72 | 36.92 | 32.64 | 12.72 |
|  | **2 2 1 1** | **0.84** | **0.77** | **0.78** | **0.83** | **-34058.01** | **-34011.55** | **24.33** | **6.63** | **5.84** | **39.89** | **17.93** | **32.83** | **36.67** | **12.58** |
|  | 1 1 1 1 | 0.79 | 0.82 | 0.84 | 0.77 | -34137.25 | -34097.43 | 6.09 | 35.99 | 24.36 | 6.49 | 36.50 | 12.14 | 18.08 | 33.28 |
|  | 1 1 1 0 | 0.77 | 0.79 | 0.84 | 0.82 | -34135.97 | -34099.47 | 6.46 | 6.05 | 24.54 | 38.43 | 33.51 | 36.40 | 18.16 | 11.93 |
|  | 1 1 0 0 | 0.84 | 0.76 | 0.78 | 0.83 | -34145.73 | -34112.55 | 24.30 | 6.46 | 5.86 | 40.21 | 18.10 | 32.86 | 36.81 | 12.22 |
|  | 1 0 0 0 | 0.85 | 0.77 | 0.78 | 0.81 | -34152.43 | -34122.57 | 22.84 | 6.49 | 6.02 | 39.76 | 19.81 | 34.16 | 35.13 | 10.90 |

^a^ 0=intercept, 1=linear, 2=quadratic, 3=cubic

Notes: AvePP, average posterior probability; BIC, Bayesian information criterion; AIC, Akaike information criterion; OCC: odds of correct classification. Finally, we chose the trajectory model of the four groups (Order: 2 2 1 1)

**Table S2.** The final four-group trajectory model of cognitive scores from 2011-2020 in CHARLS.

| Trajectory group | Parameter | Maximum likelihood estimates | | | |
| --- | --- | --- | --- | --- | --- |
|  |  | Est. | SE | Z value | P |
| Class 1: low and decline  (n = 1003, 17.9%) | Intercept | 55085.87259 | 393.83581 | 139.870 | <0.0001 |
|  | Linear | -54.51729 | 0.05290 | -1030.513 | <0.0001 |
|  | Quadratic | 0.01349 | 0.00010 | 136.673 | <0.0001 |
| Class 2: low and stable  (n = 1856, 32.8%) | Intercept | 64321.07014 | 177.34257 | 362.694 | <0.0001 |
|  | Linear | -63.80151 | 0.02579 | -2473.808 | <0.0001 |
|  | Quadratic | 0.01582 | 0.00004 | 353.860 | <0.0001 |
| Class 3: middle and stable  (n = 2157, 36.7%) | Intercept | -35.07194 | 7.37269 | -4.757 | <0.0001 |
|  | Linear | 0.01761 | 0.00366 | 4.812 | <0.0001 |
| Class 4: high and stable  (n = 621, 12.6%) | Intercept | -27.00549 | 11.47817 | -2.353 | 0.0186 |
|  | Linear | 0.01409 | 0.00570 | 2.474 | 0.0134 |

Notes: Est, parameter estimate; SE, standard error of parameter estimate.

**Table S3.** Characteristics of participants by the cognitive trajectory.

| Characteristic | High and stable (n=621) | Middle and stable (n=2157) | Low and stable (n=1856) | Low and decline (n=1003) | P value |
| --- | --- | --- | --- | --- | --- |
| Age, n (%) |  |  |  |  | <0.001 |
| 45-59 | 453 (72.9) | 1289 (59.8) | 899 (48.4) | 325 (32.4) |  |
| >=60 | 168 (27.1) | 868 (40.2) | 957 (51.6) | 678 (67.6) |  |
| Sex, n (%) |  |  |  |  | <0.001 |
| Male | 303 (48.8) | 1238 (57.4) | 1009 (54.4) | 458 (45.7) |  |
| Female | 318 (51.2) | 919 (42.6) | 847 (45.6) | 545 (54.3) |  |
| Education, n (%) |  |  |  |  | <0.001 |
| Elementary school or below | 134 (21.6) | 981 (45.5) | 1367 (73.7) | 929 (92.6) |  |
| Secondary school | 435 (70.0) | 1131 (52.4) | 482 (26.0) | 74 ( 7.4) |  |
| College and above | 52 ( 8.4) | 45 ( 2.1) | 7 ( 0.4) | 0 ( 0.0) |  |
| Residence, n (%) |  |  |  |  | <0.001 |
| Rural | 337 (54.3) | 1580 (73.2) | 1607 (86.6) | 913 (91.0) |  |
| City | 284 (45.7) | 577 (26.8) | 249 (13.4) | 90 ( 9.0) |  |
| Marital status, n (%) |  |  |  |  | <0.001 |
| No | 27 ( 4.3) | 143 ( 6.6) | 178 ( 9.6) | 175 (17.4) |  |
| Yes | 594 (95.7) | 2014 (93.4) | 1678 (90.4) | 828 (82.6) |  |
| Smoking, n (%) |  |  |  |  | <0.001 |
| No | 399 (64.3) | 1211 (56.1) | 998 (53.8) | 608 (60.6) |  |
| Yes | 222 (35.7) | 946 (43.9) | 858 (46.2) | 395 (39.4) |  |
| Drinking, n (%) |  |  |  |  | <0.001 |
| No | 379 (61.0) | 1300 (60.3) | 1183 (63.7) | 688 (68.6) |  |
| Yes | 242 (39.0) | 857 (39.7) | 673 (36.3) | 315 (31.4) |  |
| BMI, n (%) |  |  |  |  | <0.001 |
| Normal | 287 (46.2) | 1105 (51.2) | 1014 (54.6) | 576 (57.4) |  |
| Underweight | 20 ( 3.2) | 70 ( 3.2) | 112 ( 6.0) | 88 ( 8.8) |  |
| Overweight | 217 (34.9) | 718 (33.3) | 534 (28.8) | 243 (24.2) |  |
| Obesity | 97 (15.6) | 264 (12.2) | 196 (10.6) | 96 ( 9.6) |  |
| WC, n (%) |  |  |  |  | 0.013 |
| Normal | 463 (74.6) | 1674 (77.6) | 1429 (77.0) | 729 (72.7) |  |
| Abdominal obesity | 158 (25.4) | 483 (22.4) | 427 (23.0) | 274 (27.3) |  |
| TG, mean (SD) | 1.33 (0.64) | 1.31 (0.64) | 1.26 (0.61) | 1.24 (0.58) | 0.001 |
| HDL, mean (SD) | 1.31 (0.36) | 1.33 (0.37) | 1.37 (0.37) | 1.39 (0.38) | <0.001 |
| Cognitive score, mean (SD) | 22.16 (2.70) | 18.24 (2.97) | 15.58 (2.98) | 13.22 (2.52) | <0.001 |
| VAI, n(%) |  |  |  |  | 0.092 |
| Q1 | 138 (22.2) | 520 (24.1) | 484 (26.1) | 268 (26.7) |  |
| Q2 | 143 (23.0) | 536 (24.8) | 477 (25.7) | 253 (25.2) |  |
| Q3 | 160 (25.8) | 537 (24.9) | 467 (25.2) | 245 (24.4) |  |
| Q4 | 180 (29.0) | 564 (26.1) | 428 (23.1) | 237 (23.6) |  |

Notes: BMI, body mass index; WC, waist circumference; TG, triglycerides; HDL, high-density lipoprotein cholesterol; VAI, visceral adiposity index; SD, standard deviation.

**Table S4.** Multinomial logistic regression analysis of the relationship between VAI quartile and cognitive trajectories (N = 3966)**.**

| VAI | Middle and stable ^a^ | | Low and stable ^a^ | | Low and decline ^a^ | |
| --- | --- | --- | --- | --- | --- | --- |
|  | OR (95% CI) ^b^ | *P* | OR (95% CI) ^b^ | *P* | OR (95% CI) ^b^ | *P* |
| Q1 | Ref | | Ref | | Ref | |
| Q2 | 1.29 (0.95,1.77) | 0.106 | 1.15 (0.83,1.61) | 0.405 | 1.12 (0.75,1.68) | 0.585 |
| Q3 | 1.03 (0.76,1.39) | 0.854 | 0.84 (0.61,1.16) | 0.283 | 0.77 (0.52,1.16) | 0.211 |
| Q4 | 1.14 (0.84,1.55) | 0.392 | 0.80 (0.58,1.12) | 0.196 | 0.64 (0.42,0.97) | 0.034 |

^a^ reference group was the high and stable group.

^b^ adjusted for age, sex, education, residence, marital status, smoking status, and drinking status.

Abbreviations: Ref, reference; OR, odds ratio; CI, confidence interval; VAI, visceral adiposity index.
